# A Novel Target Pathogen Identification and Tracking System Using Capillary Electrophoresis-Random Amplified Polymorphic DNA

**DOI:** 10.1038/s41598-018-33702-6

**Published:** 2018-10-18

**Authors:** Wei-Ju Lin, Chien-Yi Tung, Muh-Yong Yen, Yu-Jiun Chan, Chi-Hung Lin, Po-Ren Hsueh

**Affiliations:** 1 Institute of Microbiology and Immunology, National Yang-Ming University, Taipei, 11221 Taiwan; 2 VYM Genome Research Center, National Yang-Ming University, Taipei, 11221 Taiwan; 3 Division of Infectious Disease, Taipei City Hospital, Taipei, 10844 Taiwan; 4 School of Medicine, National Yang-Ming University, Taipei, 11221 Taiwan; 5 Institute of Public Health, National Yang-Ming University, Taipei, 11221 Taiwan; 6 Division of Infectious Diseases, Department of Medicine, Taipei Veterans General Hospital, Taipei, 11217 Taiwan; 7 Division of Microbiology, Department of Pathology and Laboratory Medicine, Taipei Veterans General Hospital, Taipei, 11217 Taiwan; 8 Departments of Laboratory Medicine and Internal Medicine, National Taiwan University Hospital, Taipei, 10617 Taiwan

## Abstract

Rapid and accurate identification of pathogen is a major quarantine strategy for outbreak prevention. We used capillary electrophoresis-random amplified polymorphic DNA (CE-RAPD) to generate highly discriminatory pathogen profiles, reduced batch effects between profiles by novel normalization procedure and pattern of technical repeats, followed by target similarity evaluation using target identification score (TIS). A full target signature contains several patterns. TIS system was optimized by training set isolates that included three species, and validated using two hundred clinical *Klebsiella pneumoniae* isolates. Hierarchical clustering analysis showed CE-RAPD profiles arrange clusters according to the species or the source. Moreover, samples with similar profile may display similar antibiotic susceptibility. By using a signature of four patterns, the TIS system could accurately identify target among different isolates. The variation between isolates may be caused by small change in genome. TIS system provides a standardized tool for building of outbreak firewall and facilitate data exchange.

## Introduction

Frequent international travel and increasing population density in cities greatly speed up the transmission of infectious diseases. An emerging disease can spread globally within days. Drawing from experience with SARS and *Neisseria meningitidis*^1,2^, immediate and precise quarantine is the most effective defense. An accurate, rapid and convenient method of pathogen identification becomes the key to immediate quarantine and routine tracking of transmissions^3^. Pathogen identification is also important for disease treatment and infection control.

Several molecular methods are used for identification of pathogens^4,5^. Restriction fragment length polymorphism (RFLP) which analyzes the polymorphism of digested genomic fragments as the specific fingerprint of microbial by pulsed-field gel electrophoresis (PFGE), is the gold standard for microbial typing^6^. However, RFLP-PFGE requires large sample input and takes longer to run, thus limiting its application in rapid and large-scale screening^7^. Multilocus sequence typing (MLST) is another common method, which uses sequence of specific housekeeping genes. The result is easily comparable between laboratories^8^. RFLP and MLST are both the kinds of signature-base identification methods. Various other methods have also been developed, including short-sequence DNA repeats (SSRs), multiplex PCR and matrix-assisted laser desorption/ionization time-of-flight mass spectrometry (MALDI-TOF MS)^7,9^. But, these methods are limited in identification of unknown microbe because of the requirement of specific primer or predefined database and low discrimination depth^7,9,10^.

Random amplified polymorphic DNA (RAPD) PCR amplify genome by using 8–12 bp length primers, and generates highly polymorphic fragments profiles as microbial identification fingerprints^11^. All RAPD primers can amplify using the same PCR program and the amplicons are usually smaller than 3 kb. Profiles can be rapidly analyzed without time-consuming PFGE^12^. Although, RAPD presents problems of reproducibility between batches^7^, several normalization and standardization algorithms have been developed to overcome this problem^13,14^.

We developed a RAPD-based pathogen identification system by using capillary electrophoresis (CE) instead of agarose gel electrophoresis (AGE)^15^. High sensitivity of CE greatly improved resolution of small molecular detection in RAPD profiling and also reduced sample input requirement^16^. We developed a novel normalization and scoring procedure to identify target profiles from signal variation and batch effects. A set of RAPD patterns were combined to create a signature for identifying target sample. The standardized data procedure facilitates CE-RAPD result exchange between laboratories. The digital signature of pathogens can be broadcast through the internet, which helps to establish a rapid transmission defense system to block infectious disease outbreaks. By digitizing the RAPD profile, information is made accessible, thus enabling all healthcare members to have immediate access to consistent information to build a protective wall against the disease.

## Results

### Reproducibility and resolution of CE-RAPD

The reproducibility of CE-RAPD was tested by six independent technical repeats of *Escherichia coli* isolate E18 with primer P1254, and the result was compared with conventional AGE. Bands from CE and AGE were automatically marked (White spots in Supplementary Fig. S1) using the imaging software gelanalyzer2010a (http://www.gelanalyzer.com/)^17^. Although mild shift of bands between repeats was observed, most bands were reproducibly detected in all repeats using both AGE and CE. The number of bands in CE was more than AGE; 12 bands were visualized between the 100–1500 bp region using CE, while only 7 bands were visualized using AGE. In the low molecular weight (MW) region (<500 bp), CE was more sensitive than AGE. Six consistent and minor bands (Blue arrow) were detected only in CE. In the high density peak region (500~700 bp), CE had higher resolution (4 bands) than AGE (2 bands). Our results showed that RAPD were reproducible using both AGE and CE. However, CE showed higher resolution than AGE, especially in low-MW and high density peak regions that are common variant properties between samples. We further tested the sensitivity of CE-RAPD by using serial diluted samples. The results showed the minimal sample input was 10^5^ CFU (about 100 pg genomic DNA) (Supplementary Fig. S2).

### Data processing of CE-RAPD profiles

**Figure 1:**
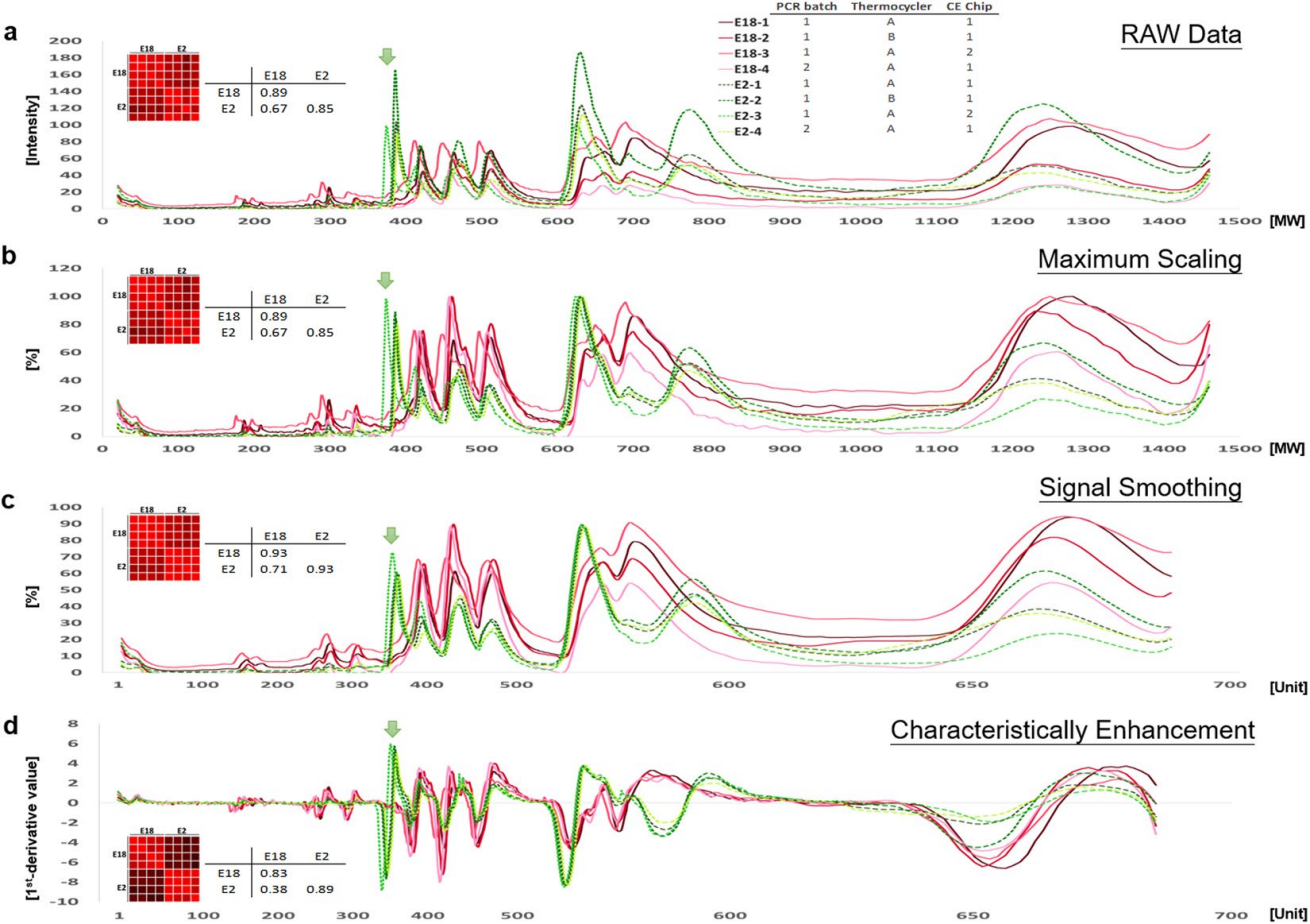
Capillary electrophoresis (CE) data processing flowchart. Red lines represent isolate E18, while green lines represent isolate E2. CE-RAPD profiles of four technical repeats were generated from two PCR batch reactions, two thermocyclers and two different CE chips (shown in top Table). The heat map and correlation coefficient (r) were derived using Pearson’s correlation analysis. (**a**) CE-RAPD profiles RAW data. (**b**) Maximum signal scaling. (**c**) Whole signal smoothing processing. (**d**) Profile characteristically enhancement.

We demonstrated the data processing workflow with two *E*. *coli* isolates (E2 and E18). Four CE-RAPD profiles of each isolate were collected under different conditions. Band shifts and intensity variation were observed between repeats (Green arrows, Fig. 1a). The intergroup correlation (E2 vs. E18 = 0.667) was close to intragroup correlations (E2: 0.854, E7: 0.887) due to these noises. We reduced intragroup variance and enhanced the intergroup difference by a three-step normalization procedure.

#### Step 1: Maximum scaling

First, we scaled the maximum intensity of each profile to 100. The scaling reduced global intensity difference between experiments (Fig. 1b), which greatly help in the following steps.

#### Step 2: Signal smoothing

Non-linear band shift of electrophoresis had been observed previously^18^. Such shifts were hard to correct by simple signal scaling. We used centered moving averages with a window of eleven to reduce the effects of the shifts. The global correlation between samples was increased after signal smoothing (Fig. 1c).

#### Step 3: Characteristically enhancement

We used the first derivative to enhance major peaks after signal smoothing^19^. This step greatly enhanced the sample characteristics. The correlation coefficient of intergroup was reduced from 0.71 to 0.38 without significant change in intragroup (Fig. 1d).

### Inter-species discrimination power of CE-RAPD

**Figure 2:**
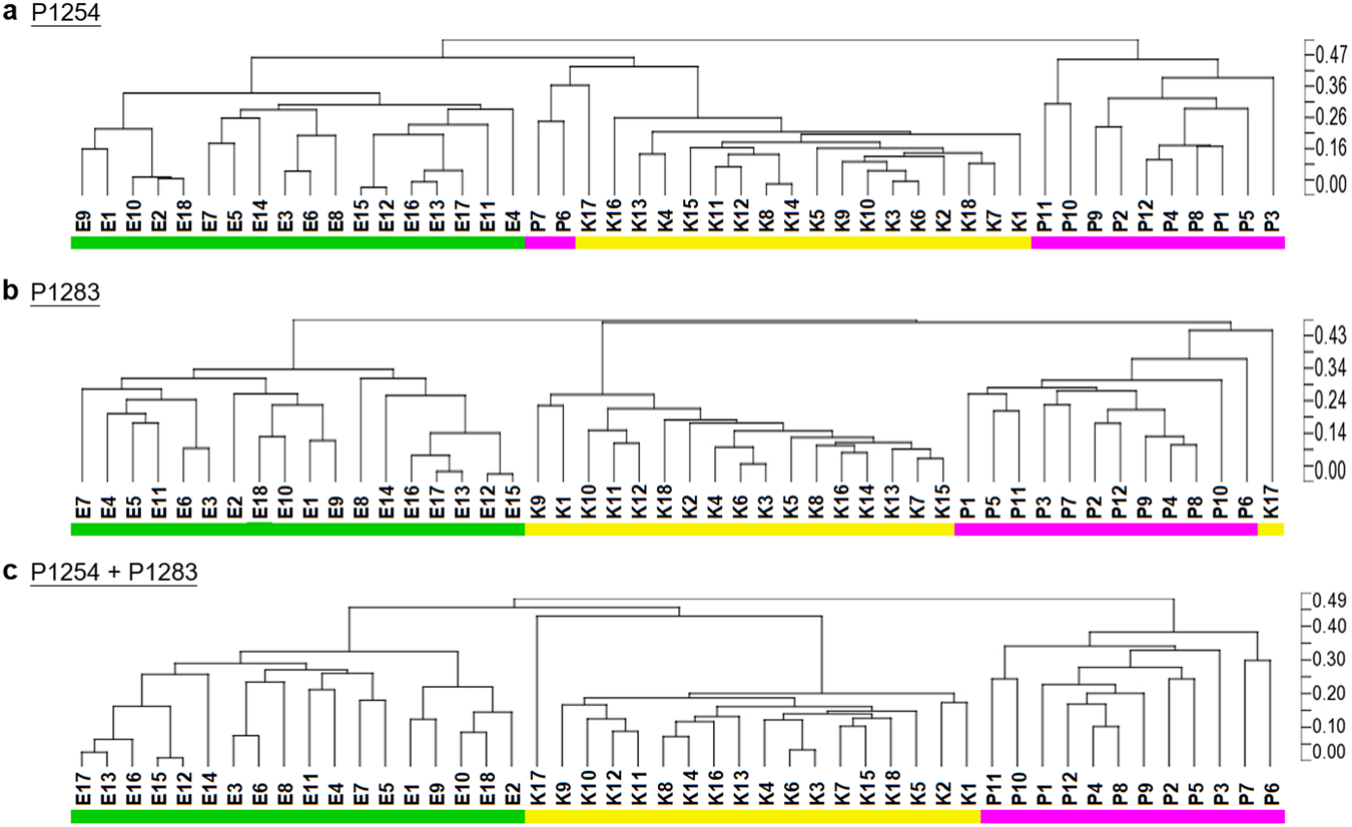
Hierarchical Clustering Analysis (HCA) was used to analyze the similarity of profiles of 48 isolates generated using primers P1254 and P1283. After data processing procedure, HCA clustering of profiles were executed. (**a**) P1254 only. (**b**) P1283 only. (**c**) Merged of P1254 and P1283. Green bar: *E*. *coli* isolates, yellow bar: *K*. *pneumoniae* isolates, and pink bar: *P*. *aeruginosa* isolates. The dissimilarity analysis were performed using Spearman dissimilarity (Partek).

We tested the data processing by using training set isolates (Fig. 2). Results showed that only one or two isolates were mis-clustered into other species in each primer. Two *Pseudomonas aeruginosa* isolates (P6 and P7) were mis-clustered into *Klebsiella pneumoniae* in P1254 primer (Fig. 2a). Isolate K17 was wrongly clustered as *P*. *aeruginosa* in primer P1283 (Fig. 2b). All isolates were successfully correctively grouped into their species cluster by merging two primer profiles of each isolate (Fig. 2c). The results show that CE-RAPD and our data procedure can correctly recognize genome character of microbial. The discrimination power of CE- RAPD profile will be improved by merging profiles of multiple primers.

### Target identification scoring system

**Figure 3:**
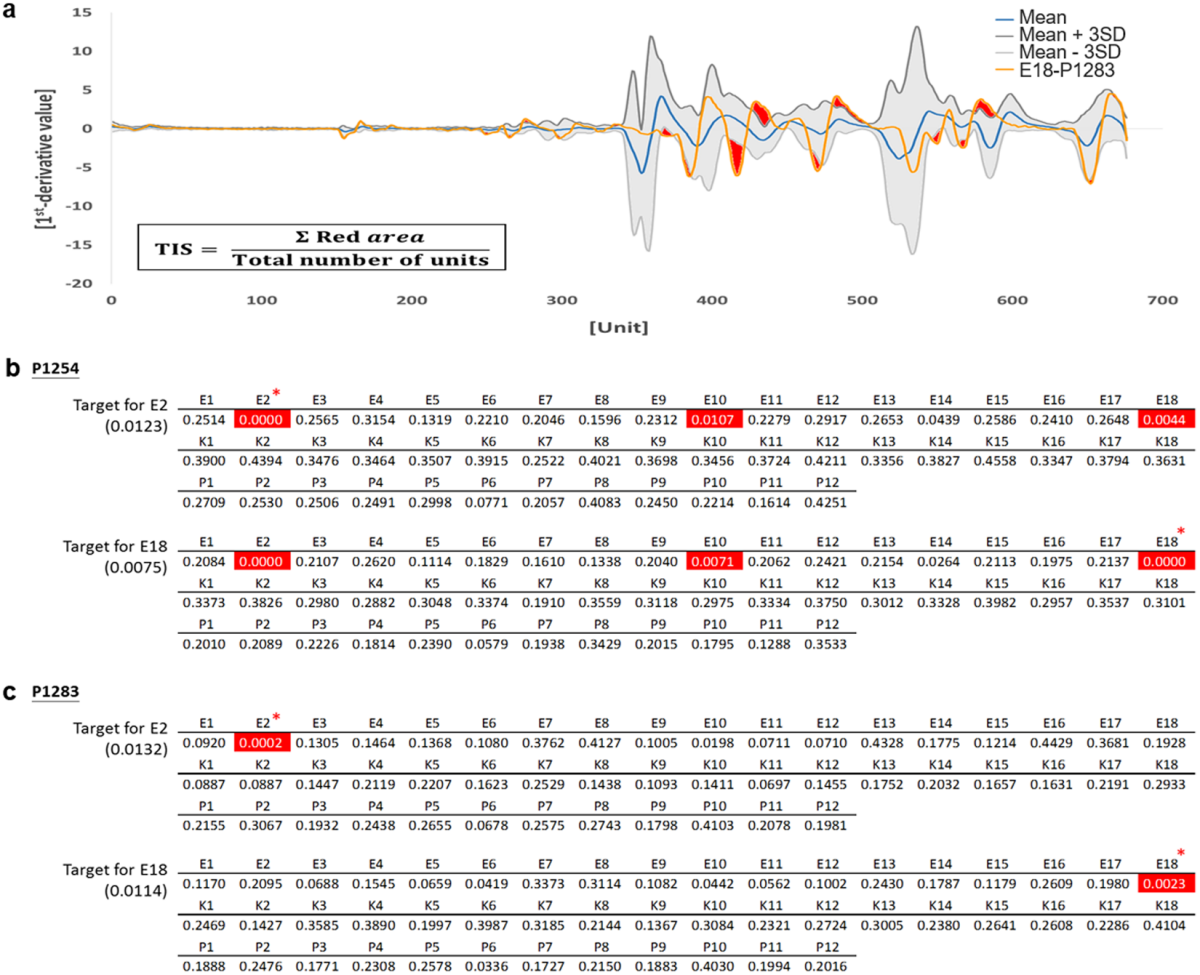
Establishing the target identification scoring system. (**a**) Schematic display of the generation of specific target pattern using E2-P1283 as model. The blue line is the mean of target profiles. The orange line is a profile of test sample, E18-P1283. The gray zone represents identification tolerance boundary. The red area represents major differences between target and test sample. The TIS formula is shown in the bottom left. (**b**,**c**) TIS system was validated by using a training set. Isolates E2 and E18 were used to assess target identification accuracy by 2 different primers, (**b**) P1254 and (**c**) P1283. The TIS thresholds for each test are shown in parentheses. Numbers highlighted in red indicate instances where the TIS was less than the threshold, in which the corresponding samples were matched to the target cluster.

We created specific target pattern from series profiles of technical repeats (see methods). Figure 3a shows an example pattern of thirty E2-P1283 profiles. The standard pattern was defined as the mean signal of the repeats (Blue line). High deviation were observed in several regions (e.g., 340–380 and 500–550 units), and each combination of primer and target have its specific deviation region (Supplementary Fig. S3). Because signals in these regions were highly variable, we defined a threshold (weighted-SD of repeats) in target pattern (Gray zone) to gate possible noise. Signals of test sample (Orange line) that exceeded the gray zone (Red area) were considered as significantly different from the target pattern. We used target identification score (TIS) to quantify profile matching of test sample with target. Briefly, TIS is the mean of signals that exceeded from the tolerance boundary (for detail see methods). When TIS of sample is smaller than threshold, it was considered identical with target. The TIS threshold was defined as “cut-off factor × maximum TIS of target repeats”. Higher cut-off factor would increase the stringency of profile identification. We evaluated TIS score with training set. E2 and E18 (Marked in*) as target were used for optimizing the weight factor of the tolerance and cut-off factor of the threshold (Supplementary Tables S1 and S2). The best positive predictive value (PPV) was found when weight factor = 3 and cut-off factor = 1.5. Discrimination accuracy of P1254 and P1283 were 95.83% and 100%, respectively (Fig. 3b,c). We used this setup in following analysis.

### Validation of the TIS system using clinical samples

**Figure 4:**
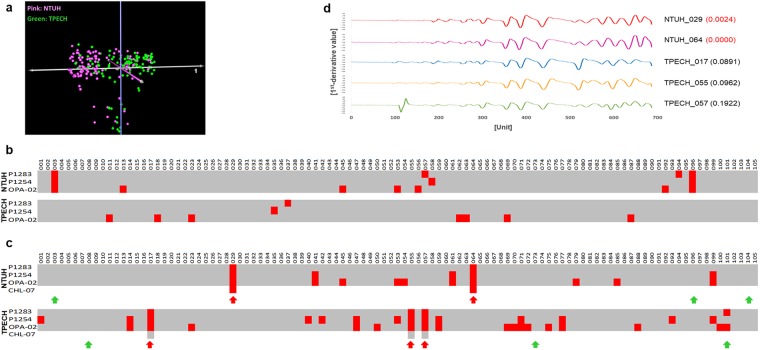
Clinical sample validation. (**a**) PCA analysis was performed on CE-RAPD profiles of 210 *K*. *pneumoniae* samples from two hospitals using combination of three primers: P1283, P1254 and OPA-02. Pink indicate samples from NTUH, and green represent samples from TPECH. (**b**) The accuracy of the TIS system was assessed using a target pattern generated using NTUH_003. The target thresholds used for each primer are as follows: P1283: 0.0048, P1254: 0.0059 and OPA-02: 0.0173. Red mark represented the TIS of test sample was matched with target. PPV of TIS system was 100%. (**c**) The accuracy of the TIS system was assessed using a target pattern generated using NTUH_029. The target thresholds used for each primer are as follows: P1283: 0.0063, P1254: 0.0193, OPA-02: 0.0326 and CHL-07: 0.0092. Arrows indicate samples that were selected for further methods comparison analysis. Red arrows indicates target matched clusters as analyzed for three primers. Green arrows indicates target mismatched clusters. PPV of TIS system were 40% (three primers) and 100% (four primers). (**d**) Visualization of CE-RAPD profile similarity and identification of target cluster after addition of the extra primer, CHL-07. Samples were NTUH_029 matched cluster from three primer analysis. The TIS of samples are shown in brackets. Red font indicates match with the target, NTUH_029.

Multidrug-resistant (MDR) bacteria are considered to be one of the most important current public health problems. *K*. *pneumoniae* is a common MDR species^20^. Transmission routine of *K*. *pneumoniae* is difficult to track because conventional microbial typing does not have enough resolution to distinguish isolates^21^. We collected 200 independent clinical *K*. *pneumoniae* isolates from two hospitals. CE-RAPD profiling was performed on a total of 210 samples including 10 duplicates. Profiles were broadly clustered into two groups according to their hospital source in Principal Component Analysis (PCA) plot (Fig. 4a). Profiles of all duplicated pairs also showed high similarity (r = 0.87~0.97, Supplementary Fig. S4), while the average coefficient of non-duplicate isolates was 0.44. We selected two isolates (low antibiotic susceptible: NTUH-003 and high antibiotic susceptible NTUH-029) as tracking target. The specific target signature consisted of three CE-RAPD patterns (P1283, P1254 and OPA-02). TIS of each sample were calculated and identified. NTUH-003 and its duplicate (NTUH-096) were perfectly identified from other isolates (Fig. 4b). In NTUH-029 cases, itself and its duplicate (NTUH-064) were also correctly identified, but three others (TPECH-017, TPECH-055 and TPECH-057) could not be excluded (Fig. 4c). We added an extra primer, CHL-07, to improve the signature resolution (Fig. 4c,d). The result demonstrated combined patterns of target signature could correctly identify target among 200 isolates by using up to four patterns signature of TIS system.

### Comparison between CE-RAPD, MLST and RFLP-PFGE

**Figure 5:**
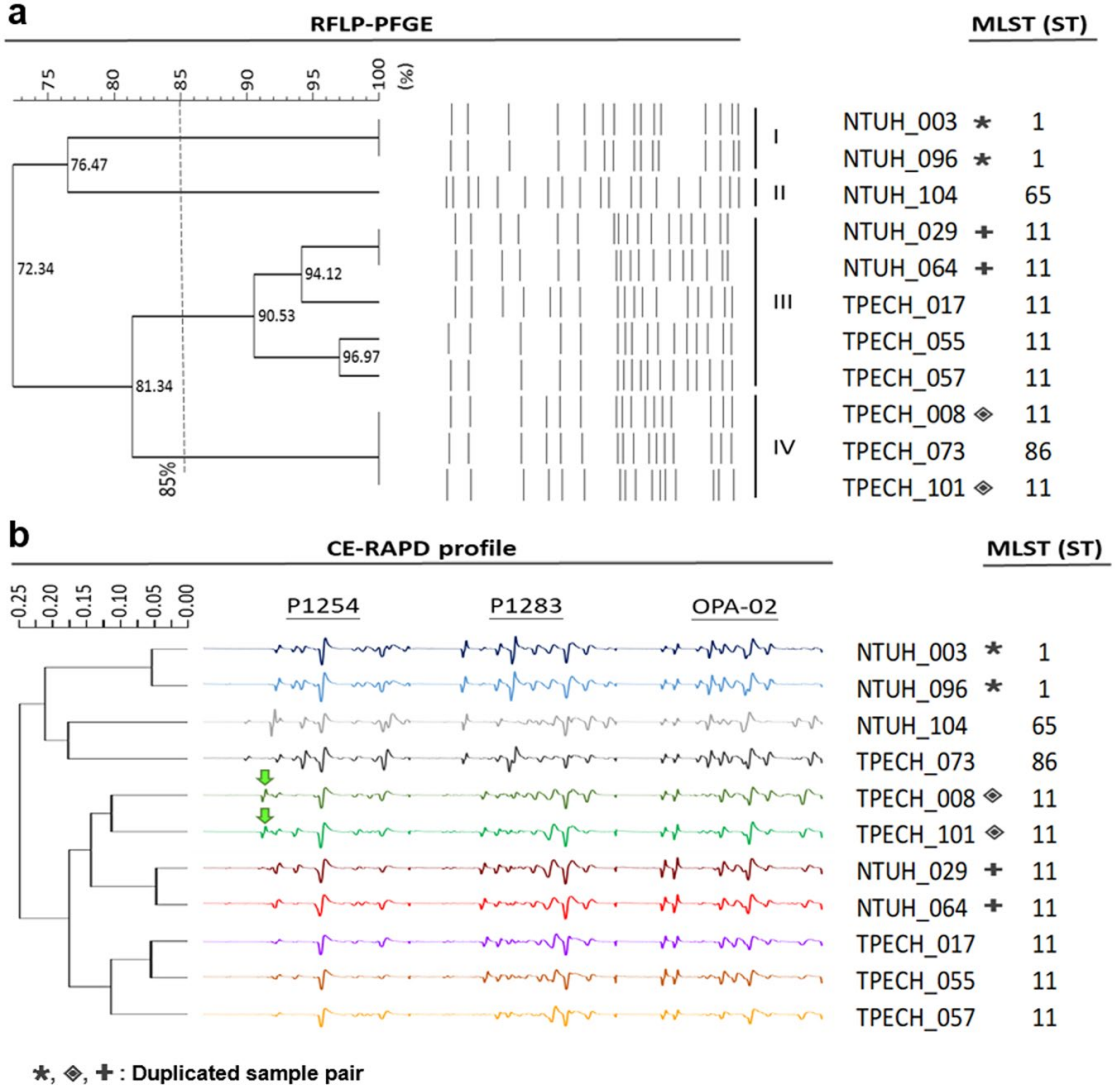
Grouping of NTUH_029 matched and mismatched samples. (**a**) RFLP-PFGE classification compared with MLST. The RFLP pattern dendrogram was constructed using the Dice coefficient (1% optimization and 1.5% position tolerance) in BioNumerics software (Applied Maths). Percentages indicate the pattern similarity. All RFLP patterns were grouped into four clusters (cut off: 85%). (**b**) Clustering of CE-RAPD profiles was compared with MLST. HCA was performed on processed profiles from the three primers, P1283, P1254 and OPA-02 using Spearman dissimilarity (Partek). Numbers beside the tree indicate the dissimilarity distance between samples profile. Green arrows point to the site of the extra amplicon in TPECH_008/101 that did not appear in the NTUH_029 matched cluster. The six mismatched samples included TPECH_008 and TPECH_101 (high antibiotic resistance); NTUH_003, NTUH_096 and TPECH_073 (low antibiotic resistance) and NTUH_104 (antibiotic sensitivity). Duplicate pairs are labeled with identical symbols.

We compared CE-RAPD with two well-established microbial typing methods (MLST and RFLP-PFGE). Eleven clinical *K*. *pneumoniae* samples were analyzed concurrently, which included 5 samples of NTUH-029 similar cluster (red arrows in Fig. 4c) and another 6 mismatched samples (green arrows in Fig. 4c). Three duplicated pairs were included in these samples (identical symbols in Fig. 5). As expected, duplicate samples showed the same MLST type. RFLP-PFGE classified samples generally according to their ST type, except TPECH-073 (ST86) which was mis-clustered into the ST11 group (Fig. 5a). All CE-RAPD profiles were perfectly grouped according to their ST type (Fig. 5b). However, TPECH-008/101 duplicated pair was ST11 type, but was not identified as NTUH-029 cluster in TIS system. We found an extra amplicon difference between TPECH-008/101 and NTUH-029 cluster in P1254 amplification (Arrow in Fig. 5b and Supplementary Fig. S5a). We cloned and sequenced this amplicon. The sequence matched with integrase core domain protein (accession: KP125893.1) in plasmid pHS08204 and was only detected in TPECH-008/101 by specific PCR (Supplementary Fig. S5b). Results showed that CE-RAPD is more consistent with MLST microbial typing than RFLP-PFGE. Moreover, TIS system has enough resolution to identify small difference (e.g., plasmid) between samples.

### The relationship between antibiotic resistance and CE-RAPD profiles similarity

**Figure 6:**
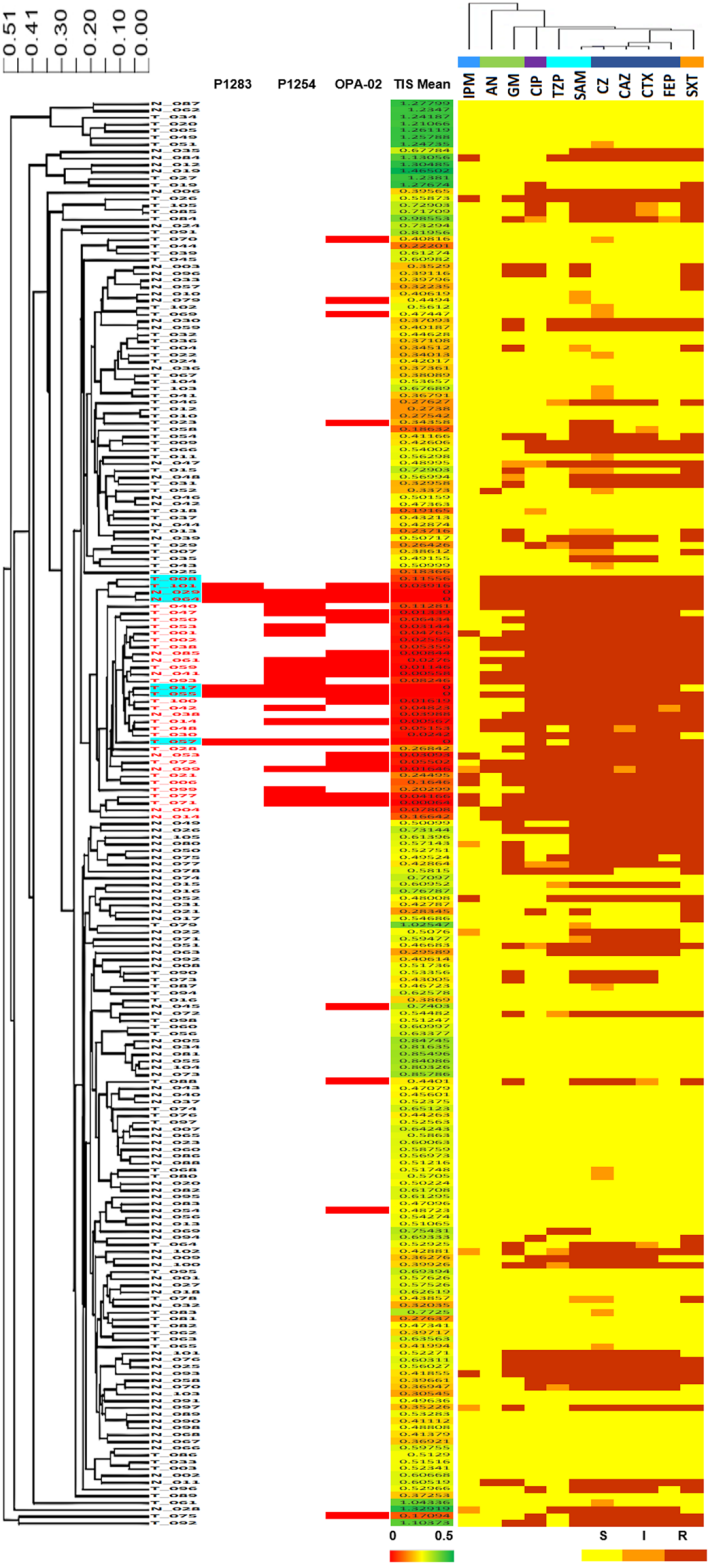
The relationship between CE-RAPD profiles, TIS correlation and antibiotic susceptibility testing (AST) pattern of 210 clinical samples. Combined clinical samples from NTUH and TPECH. HCA of CE-RAPD profiles (left side) and AST pattern (top) were performed clustering based on Spearman dissimilarity (Partek). N represents NTUH, and T represents TPECH. Red font indicates the similarly group of NTUH-029 profile (dissimilarity distance < 0.24). Blue background indicates the NTUH-029 matched cluster (Red arrows in Fig. 4c). Individual TIS identified results and the mean of TIS from three primers are shown in the center of the figure. These TIS results were target with NTUH-029. The color bar at the bottom of the figure shows the value of TIS mean, minimum 0 and maximum 0.5. Antibiotic names are indicated in the upper right-hand corner of the figure, and identical color bar indicate the same antibiotic types. IPM: imipenem. AN: amikacin. GM: gentamicin. CIP: ciprofloxacin. TZP: piperacillin-tazobactam. SAM: ampicillin-sulbactam. CZ: cefazolin. CAZ: ceftazidime. CTX: cefotaxime. FEP: cefepime. SXT: trimethoprim-sulfamethoxazole. At the bottom right of figure, color blocks indicate antibiotic susceptibility. Yellow block is susceptible (S), orange block is intermediate (I) and red block is resistance (R).

We wanted to find out whether the clustering of CE-RAPD profiles correlated with the pattern of antibiotic resistance as MLST^22^. Samples were grouped according to similarity of RADP profiles (Fig. 6). ST11 is a known common multidrug-resistant type^23^. As expected, high antibiotic susceptible isolates (ST11 group in Fig. 5b) were grouped in the same cluster (Fig. 6, blue background). Besides, 29 other samples were also grouped into this cluster (dissimilarity distance < 0.24, red font). All these samples showed similar antibiotic susceptibility pattern, and their averaged TIS were closed to NTUH-029 (Fig. 6).

## Discussion

Ongoing global ecological change will continue to produce new infectious pathogens. Effective surveillance and quarantine are important strategies to prevent emerging infectious disease outbreak^24,25^. Our TIS system offers a rapid tool to identify and track pathogen transmission after pathogens are identified. CE-RAPD not only provide high-resolution fingerprints but also can be done much faster than RFLP-PFGE^26^. It reduced the experimental time from one day to six hours. It does not rely on different restriction enzyme reaction condition or predefined database as with RFLP or MALDI-TOF MS. All primers of RAPD reaction under the same condition, and parallel combined of several patterns could extensively improve the discriminatory power of RAPD. In our study, CE-RAPD successfully identified target isolates from 200 clinical *K*. *pneumoniae* isolates by using up to four primers.

As with RFLP-PFGE, poor reproducibility between experiments is also a common problem in RAPD analysis^27,28^. The minor band absences or shifts observed in RAPD amplifications greatly affected the accuracy of pathogen identification. We developed a novel data processing method to solve the problem. Our three-step normalization process reduce batch effects but retains the profiles characteristic. It facilitated profiles standardization to assist data exchanges and comparison across institutions. In our analysis, technical repeats presented high correlation (r > 0.83) through data adjustment.

**Figure 7:**
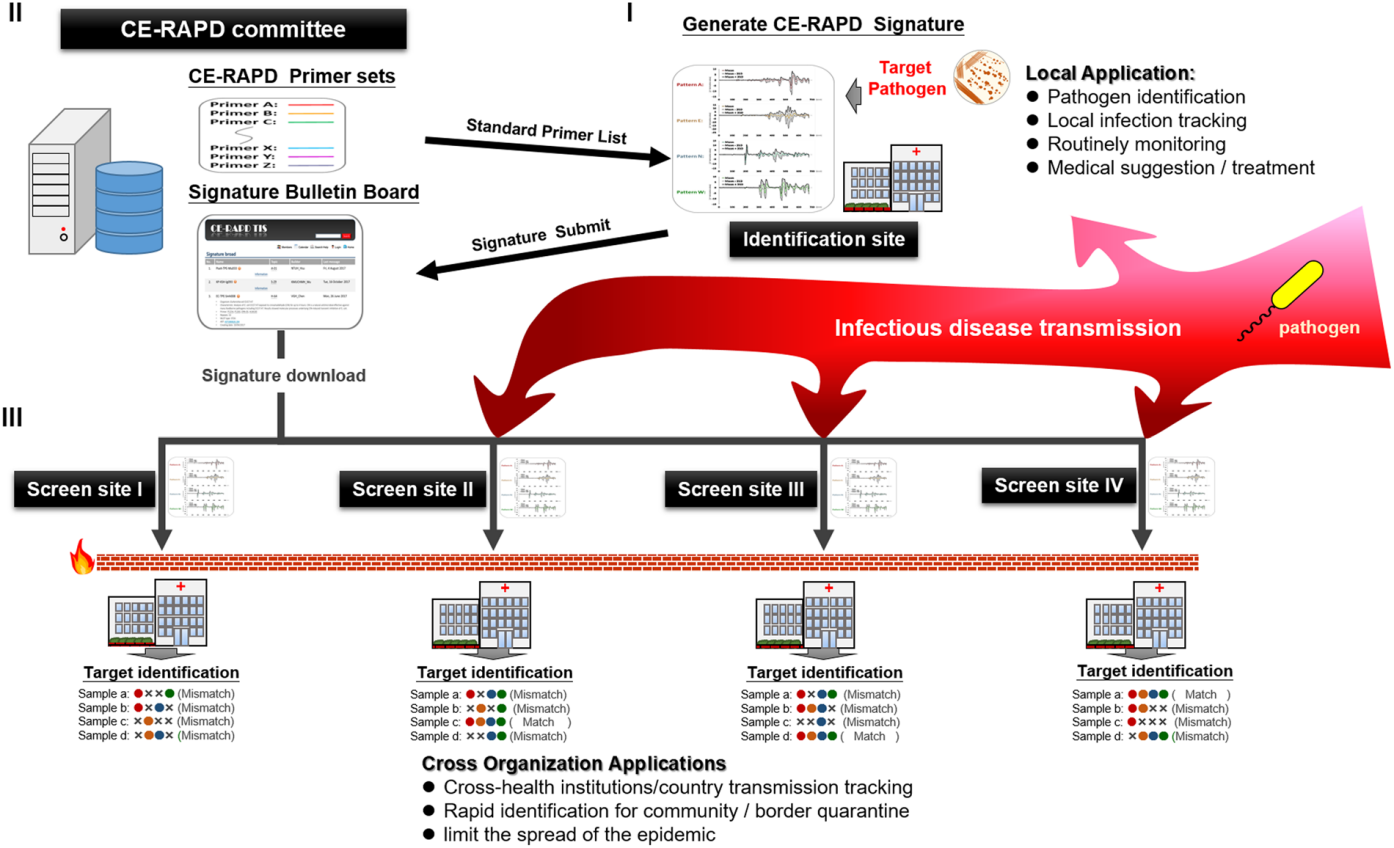
Rapid infectious disease defense system workflow. The firewall label indicates blocking and isolation of pathogen transmission.

We developed a standard data procedure to generate the independent recognizing pattern and TIS scoring to identify target. It estimated signal pattern from series of technical repeats and defined highly deviated region to gate noise. TIS can indicate whether test sample is identical to the target without requirement of reference or database. TIS system used digitized signal signature to replace complex image profiles, which facilitates rapid exchange for global disease control. Therefore, we propose a rapid infectious disease defense system based on TIS system (Fig. 7). An official committee maintains a set of standard primers for all signatures design. The standard primers will guarantee the signature uniformity for data circulation world-wide. This system will block pathogen spread through three steps as follows: (I) Target signature generation: At the first identification site, the target signature of emerging pathogen can be generated by using standard primers. The digitized signature will be submitted through the internet immediately. (II) Signature broadcast: Submitted signature will be published in the committee web site. Members of the committee can exchange information and characteristic of pathogens on the bulletin board. (III) Build up an infectious firewall: When outbreak occurs, members will be alerted and will be able to immediately assemble rapid specific screening tools by downloading the target digital signature file from the committee. This rapid synchronized defense system can become a firewall that can effectively prevent the spread of infection.

The information of pathogen identification is also useful for medical decision-making. Several reports showed molecular typing result (e.g., RFLP, RAPD and MLST) of microbial geographical distribution and serogrouping might be associated with antibiotic susceptibility^22,29–31^. But, these associations do not reflect the correlation with virulence gene of multidrug resistance strains^32,33^. Similarly, our results showed association between CE-RAPD profile and antibiotic susceptibility. All NTUH-029-like isolates have similar MDR pattern. The association of antibiotic susceptibility can be easily indicated by the averaged TIS value. When MDR strain is widely profiled, it will be possible to scan thousands of antibiotic susceptibility strains immediately. It will become powerful tools for therapy decision-making.

Moreover, we found that similar isolates (NTUH-029-like isolates cluster, Fig. 6) were collected from two different hospitals in Taipei. Taipei is one of the world’s most densely populated city, where cross-infection between persons can easily occur^34^. This example illustrates CE-RAPD is a potential tool for detecting cross-region transmission.

Though we have demonstrated the advantages of the TIS system in infectious disease prevention, some questions remain to be solved. Although batch effects of different instrument, enzymes or reagents were reduced by data normalization, it is still a big challenge to compared data from laboratories around the world. An ideal solution, such as a closed microfluidic system^35^ with integrated PCR and capillary electrophoresis, which will greatly speed up processing time and increase reproducibility may be able to solve the problem. In addition, it is difficult to identify all kinds of tiny genomic differences with a single primer set, and no other pre-existing signature-based methods can solve this shortcoming except the expensive whole genome sequencing. CE-RAPD was developed as a rapid pathogen screening and identification tool in the first tier. How many primers will be necessary to provide cost-efficient resolution in real clinical practices worldwide is yet to be investigated through large-scale studies.

## Conclusion

In this study, we developed a pathogen typing system by using capillary electrophoresis-random amplified polymorphic DNA, which reduced batch effects and facilitated data exchanges between laboratories through data normalization processing. Moreover, CE-RAPD profiles similarity may application for microbial species classification and collected source clustering. By using up to four patterns, the TIS provided a tool for rapid and correct target identification among different isolates. In addition, similar CE-RAPD profiles and TIS score clustering could be applied to predict antibiotic susceptibility grouping of samples. Following above strategy, epidemic surveillance agencies can instantly obtain screening tools and build an infectious diseases firewall by downloading digital signatures for pathogen identification, which serves to prevent outbreaks effectively.

## Methods

### Collection of bacteria isolates

Training set: A total of 48 isolates including three species; *E. coli* (n = 18), *K. pneumoniae* (n = 18) and *P. aeruginosa* (n = 12); were collected from National Taiwan University Hospital (NTUH, 2900 beds) and American type culture collection (ATCC), respectively.

Test set: 200 de-identified clinical *K*. *pneumoniae* isolates were collected from NTUH (January 2014 to August 2015) and Taipei City Hospital (TPECH, 2927 beds, November 2015). Each hospital collected 100 isolates and tested their antibiotic susceptibility. Additionally, five duplicate isolates collected in each hospital were used as spike-in controls. Collection of all clinical isolates were approved by the Institutional Review Board of Taipei Veterans General Hospital (code 2013-06-019BC).

### Antibiotic susceptibility testing (AST)

The antibiotic susceptibility of all clinical *K*. *pneumoniae* isolates were determined. The AST patterns of isolates from TPECH were tested by the disk diffusion method. The AST patterns of isolates from NTUH were obtained using the commercial VITEK 2 AST system (bioMérieux, Marcy l’Etoile, France). AST patterns of the nine isolates from the disk diffusion method were futher validated using the VITEK 2 AST system.

### CE-RAPD profiling

Test samples were cultured overnight on Tryptic Soy Agar (TSA) at 37 °C. Single colony of microbial was picked and suspended in 2.4 μl of ddH_2_O, then was pre-heat-lysed at 96 °C for 13 min. The final RAPD-PCR reaction mixture contained: 6 μM random primer (Supplementary Table S3), 2 μl of 5× colorless buffer, 6 mM MgCl_2_, 0.2 mM dNTP, 2 ng of BSA (NEB), 3 U of GoTaq DNA polymerase (Promega), 2.4 μl of bacteria lysate and enough ddH_2_O for a total volume of 10 μl. PCR was performed in a thermocycler (ABI 9700, Applied Biosystems) using the following program: 96 °C denaturing step for 30 s, then two reaction cycles – 10 cycles of 96 °C for 1 s, 36 °C for 5 s and 72 °C for 15 s, and 30 cycles of 96 °C for 1 s, 57 °C for 5 s and 72 °C for 15 s. RAPD profiles were analyzed using Bioanalyzer 2100 CE system in conjunction with the DNA 1000 LabChip (Agilent).

### CE-RAPD target pattern generation

Specific CE-RAPD patterns of target from >24 technical repeats including different PCR reagent batches, mixture preparations, thermocyclers and CE-reagent lots, were evaluated. The standard pattern was defined by the mean signal of repeats. A weight factor × SD of signal defined possible diversity in each unit. The weight factor can be adjusted to change the stringency of profile matching.

### Target identification score (TIS) calculation

TIS is an average of the discriminant value $({f}_{({S}_{i})})$. Briefly, ${f}_{({S}_{{i}^{}})}$ represents the distance that is the difference between the test signal and the tolerance boundary of target pattern (weight factor × SD). The ${f}_{({S}_{{i}^{}})}$ was calculated according to the following equation:

$${f}_{({S}_{i})}=\{\begin{array}{cc}|{S}_{i}-({M}_{i}+{\rm{W}}{D}_{i})|, & {S}_{i} > {M}_{i}+{\rm{W}}{D}_{i}\\ |{S}_{i}-({M}_{i}-{\rm{W}}{D}_{i})|, & {S}_{i} < {M}_{i}-{\rm{W}}{D}_{i}\\ \,0\,, & {M}_{i}+{\rm{W}}{D}_{i} > {S}_{i} > {M}_{i}-{\rm{W}}{D}_{i}\end{array}\}$$

$${\rm{TIS}}=(\frac{{\sum }_{i=1}^{n}\,{f}_{({S}_{i})}}{n})$$

Here, *i* indicates detected unit from CE. *n* indicates the total number of units. *S* is the signal intensity of the test sample. *M* is the mean of the signal intensity in the target pattern. *D* is the signal standard variation (SD) of the target pattern. W is the SD weight factor.

### RFLP-PFGE

Genomic DNA was digested with 20 U of *Xba*I (NEB). RFLP products were resolved in 1% agarose gel using PFGE (CHEF-DR III system, Bio-Rad). PFGE was performed with linear switching time from 5 to 40 s and a constant voltage of 6 V/cm at 14 °C for 22 h. The dendrogram was analyzed by using BioNumerics v5.10 (Applied Maths).

### MLST analysis

MLST primers (Supplementary Table S3) and PCR program were used as previously described^36^. PCR product was purified using Gel/DNA Fragments Extraction Kit (Geneaid) and sequenced at the VGH Yang-Ming Genome Research Center. The alleles and sequence type (ST) were assigned using the MLST website (http://bigsdb.web.pasteur.fr).

### Detection of integrase core domain protein in plasmid

Using Plasmid Miniprep Plus Purification Kit (GeneMark) extracted plasmid DNA. PCR was used to detect gene of integrase core domain protein in the plasmid. The reaction mixture contained: primers (Supplementary Table S3) at a 200 nM final concentration, 2 μl of 5× colorless buffer, 2.5 mM MgCl_2_, 0.2 mM dNTP, 1 U of GoTaq DNA polymerase (Promega), 1 ng of plasmid DNA and enough ddH_2_O for a total volume of 10 μl. The amplification program was as follows: 96 °C denaturing step for 2 min, then 30 cycles of 96 °C for 30 s, 68.7 °C for 15 s and 72 °C for 30 s. The PCR product was electrophoresis in TBE gel, and was visualization with SYBR Safe DNA dye staining.

## Electronic supplementary material

Supplementary Data

## Data Availability

All relevant data supporting the findings of the study are available within this article and its supplementary information files, or from the corresponding author upon reasonable request.
