# Supplementary material for: A Novel Target Pathogen Identification and Tracking System Using Capillary Electrophoresis-Random Amplified Polymorphic DNA
Source: Sci Rep. 2018 Oct 18;8:15365. doi: 10.1038/s41598-018-33702-6 (PMC6193972; doi:10.1038/s41598-018-33702-6)
Supplement: Supplementary file 1 — Supplementary Data [file 41598_2018_33702_MOESM1_ESM.docx]

A Novel Target Pathogen Identification and Tracking System Using Capillary Electrophoresis-Random Amplified Polymorphic DNA

**Wei-Ju Lin^1, #^, Chien-Yi Tung^2, #^, Muh-Yong Yen^3,4^, Yu-Jiun Chan^5,6,7^, Chi-Hung Lin^1,＊^ ＆ Po-Ren Hsueh^8,＊^**

^1^Institute of Microbiology and Immunology, National Yang-Ming University, Taipei, 11221, Taiwan. ^2^VYM Genome Research Center, National Yang-Ming University, Taipei, 11221, Taiwan. ^3^Division of Infectious Disease, Taipei City Hospital, Taipei, 10844, Taiwan. ^4^School of Medicine, National Yang-Ming University, Taipei, 11221, Taiwan. ^5^Institute of Public Health, National Yang-Ming University, Taipei, 11221, Taiwan. ^6^Division of Infectious Diseases, Department of Medicine, Taipei Veterans General Hospital, Taipei, 11217, Taiwan. ^7^Division of Microbiology, Department of Pathology and Laboratory Medicine, Taipei Veterans General Hospital, Taipei, 11217, Taiwan. ^8^Departments of Laboratory Medicine and Internal Medicine, National Taiwan University Hospital, Taipei, 10617, Taiwan. **^#^**Wei-Ju Lin and Chien-Yi Tung contributed equally to this work. **^＊^**Correspondence and requests for materials should be addressed to C.-H.L. (email: [linch@ym.edu.tw](mailto:linch@ym.edu.tw)) or to P.-R.H. (email: [hsporen@ntu.edu.tw](mailto:hsporen@ntu.edu.tw))

**Supplementary Data**

**Tables**

**Supplementary Table S1.** PPV analysis of different combinations of SD weight factors and cutoff factors in a 48-sample training set using E2 as the target.

|  |  |  |  | SD weight factor | | |
| --- | --- | --- | --- | --- | --- | --- |
| **Target** | **Primer** | **Cutoff factor** |  | 1 | 2 | 3 |
|  |  |  |  |  |  |  |
| E2 | P1254 | 1 |  | 25% | 33.33% | 50% |
|  |  | 1.5 |  | 9.09% | 33.33% | 33.33% |
|  |  | 2 |  | 3.45% | 20% | 33.33% |
|  |  |  |  |  |  |  |
|  | P1283 | 1 |  | 100% | 100% | 100% |
|  |  | 1.5 |  | 50% | 100% | 100% |
|  |  | 2 |  | 7.69% | 100% | 100% |
|  |  |  |  |  |  |  |
|  | P1254 + P1283 | 1 |  | 100% | 100% | 100% |
|  |  | 1.5 |  | 50% | 100% | 100% |
|  |  | 2 |  | 12.50% | 100% | 100% |

**Supplementary Table S2.** PPV analysis of different combinations of SD weight factors and cutoff factors in a 48-sample training set using E18 as the target.

|  |  |  |  | SD weight factor | | |
| --- | --- | --- | --- | --- | --- | --- |
| **Target** | **Primer** | **Cutoff factor** |  | 1 | 2 | 3 |
|  |  |  |  |  |  |  |
| E18 | P1254 | 1 |  | 33.33% | 33.33% | 50% |
|  |  | 1.5 |  | 14.29% | 33.33% | 33.33% |
|  |  | 2 |  | 8.33% | 33.33% | 33.33% |
|  |  |  |  |  |  |  |
|  | P1283 | 1 |  | 0% | ND | 100% |
|  |  | 1.5 |  | 10% | 50% | 100% |
|  |  | 2 |  | 4.35% | 25% | 100% |
|  |  |  |  |  |  |  |
|  | P1254 + P1283 | 1 |  | 0% | ND | 100% |
|  |  | 1.5 |  | 33.30% | 50% | 100% |
|  |  | 2 |  | 11.11% | 33.30% | 100% |

**Supplementary Table S3.** Primer list

| **Gene** | **Primer** | **Sequence** | **Reference** |
| --- | --- | --- | --- |
| RAPD | | | |
|  | P1254 | CCGCAGCCAA | [^1^](#_ENREF_1) |
|  | P1283 | GCGATCCCCA | [^2^](#_ENREF_2) |
|  | OPA-02 | TGCCGAGCTG | [^3^](#_ENREF_3) |
|  | CHL_07 | ACGCAGCCAC | This study |
| MLST ^a^ | | | |
| *rpoB* | Vic3 | GGCGAAATGGCWGAGAACCA | [^4^](#_ENREF_4) |
| (1075 bp) | Vic2 | GAGTCTTCGAAGTTGTAACC |  |
| gapA | gapA173 | TGAAATATGACTCCACTCACGG |  |
| (662 bp) | gapA181 | CTTCAGAAGCGGCTTTGATGGCTT |  |
| mdh | mdh130 | CCCAACTCGCTTCAGGTTCAG |  |
| (756 bp) | mdh867 | CCGTTTTTCCCCAGCAGCAG |  |
| pgi | pgi1F | GAGAAAAACCTGCCTGTACTGCTGGC |  |
| (566 bp or more) | pgi1R | CGCGCCACGCTTTATAGCGGTTAAT |  |
|  | pgi2F (seq) | CTGCTGGCGCTGATCGGCAT |  |
|  | pgi2R (seq) | TTATAGCGGTTAATCAGGCCGT |  |
| phoE | phoE604.1 | ACCTACCGCAACACCGACTTCTTCGG |  |
| (602 bp) | phoE604.2 | TGATCAGAACTGGTAGGTGAT |  |
| infB | infB1F | CTCGCTGCTGGACTATATTCG |  |
| (462 bp) | infB1R | CGCTTTCAGCTCAAGAACTTC |  |
|  | infB2F (seq) | ACTAAGGTTGCCTCCGGCGAAGC |  |
| tonB | tonB1F | CTTTATACCTCGGTACATCAGGTT |  |
| (539 bp) | tonB2R | ATTCGCCGGCTGRGCRGAGAG |  |
| Integrase core domain protein (702 bp) | | | |
|  | KpPHS38F | GCGGGTGATTCATAGTCTAGCG | This study ^b^ |
|  | KpPHS739R | GAAGATGCATCAGGACTGGTCG |  |

1. MLST sequencing was performed using the PCR primers, except for the following two genes. For infB, primer infB2F replaced infB1F as the forward sequencing primer. In addition, the forward and reverse primers for the pgi gene were replaced with pgi2F and pgi2R, respectively.
2. Primer design target from GenBank AKA86858.1. Primer efficiency was assessed using in-silica amplification.

**Figures**

**Supplementary Figure S1. CE-RAPD profile detection efficiency compared to AGE.** Comparison of pattern reproducibility and detection resolution. R1 to R6 indicate six RAPD technical repeats. 7 and 12 are the number of bands presented in AGE or CE. White arrows point to the location of missing signals.


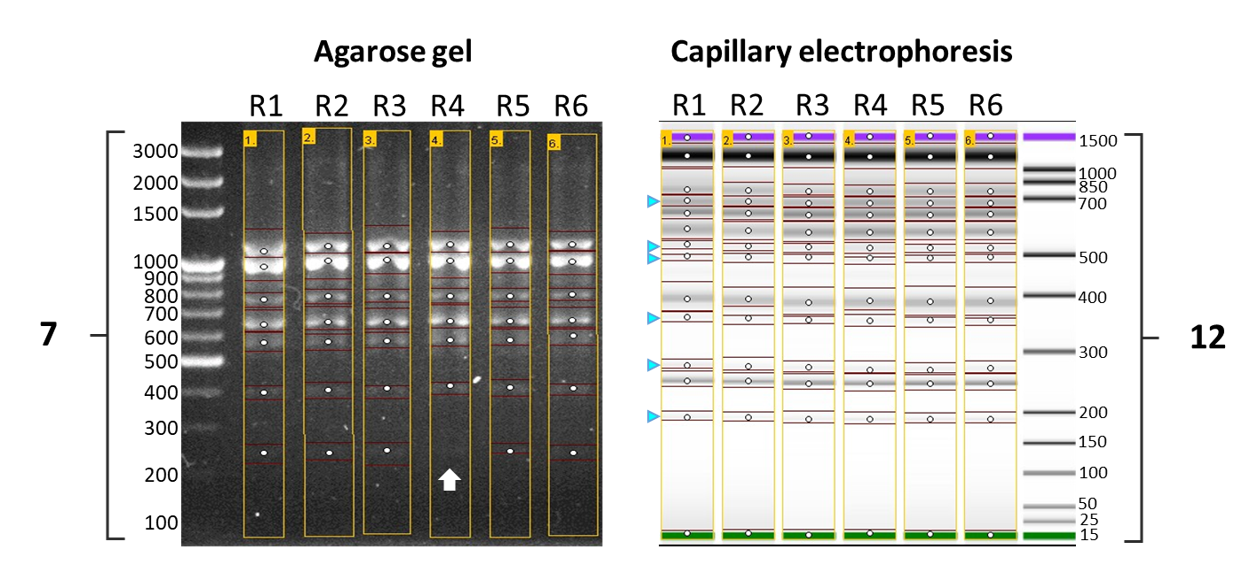


**Supplementary Figure S2.** **CE-RAPD profile sensitivity analysis.** Different bacterial counts (10 to 10^6^ CFU) or different genomic DNA mass (10 fg to 1 ng) of *E. coli* isolate E2 were used as template to amplify RAPD profiles using two primers, P1254 and P1283. (**a, c, e**) Colony RAPD. (**b, d, f**) Genomic DNA RAPD. (**a** and **b**) CE-RAPD using P1254. (**c** and **d**) CE-RAPD using P1283. (**e**) Pearson’s correlation analysis of CE-RAPD profiles was performed based on the profile of 10^6^ CFU. (**f**) Pearson’s correlation analysis of CE-RAPD profiles was performed based on the profile of 1 ng genomic DNA.


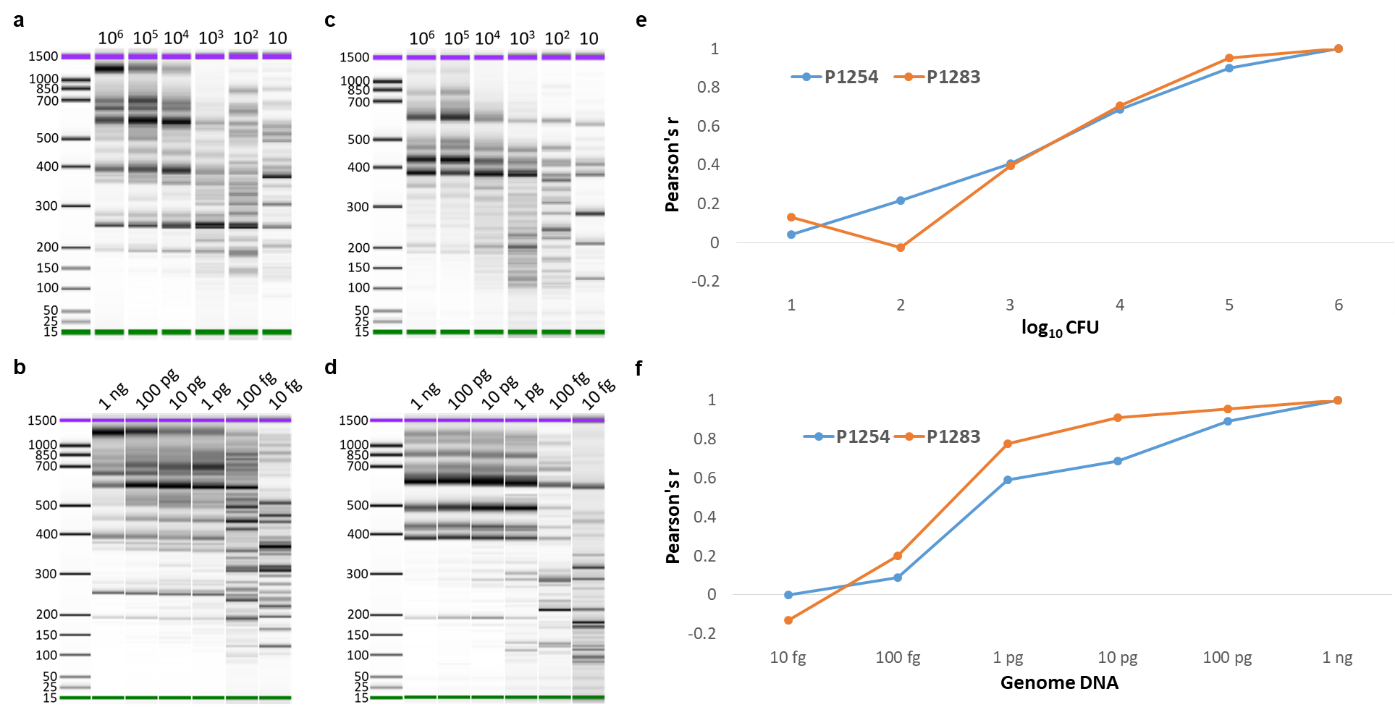


**Supplementary Figure S3.** **Specific target patterns generated using different sets of primer and target samples.** (**a**) E2-P1254. (**b**) E18-P1254. (**c**) E2-P1283. (**d**) E18-P1283.


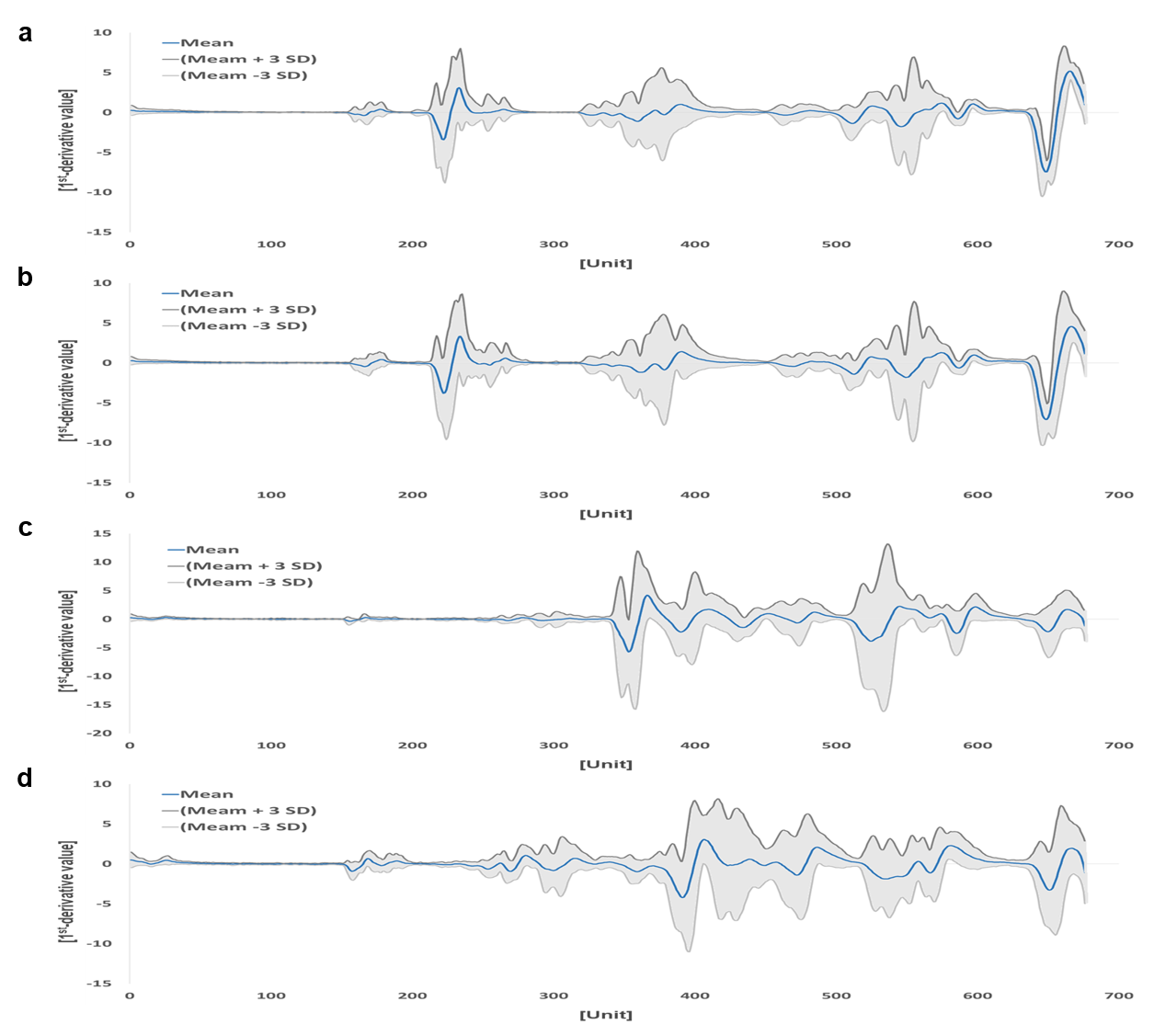


**Supplementary Figure S4.** **Pearson’s correlation analysis of CE-RAPD profiles of duplicated pairs.** (**a**) Samples from TPECH. (**b**) Samples from NTUH. Identical color indicates duplicate pairs. Pearson’s correlation was used to analyze merged CE-RAPD profiles of three primers: P1283, P1254 and OPA-02. The correlation coefficients for each duplicate pair are shown in brackets. Simulation gel of CE are shown in the upper panel, while the profiles generated after data processing are shown in the lower panel.


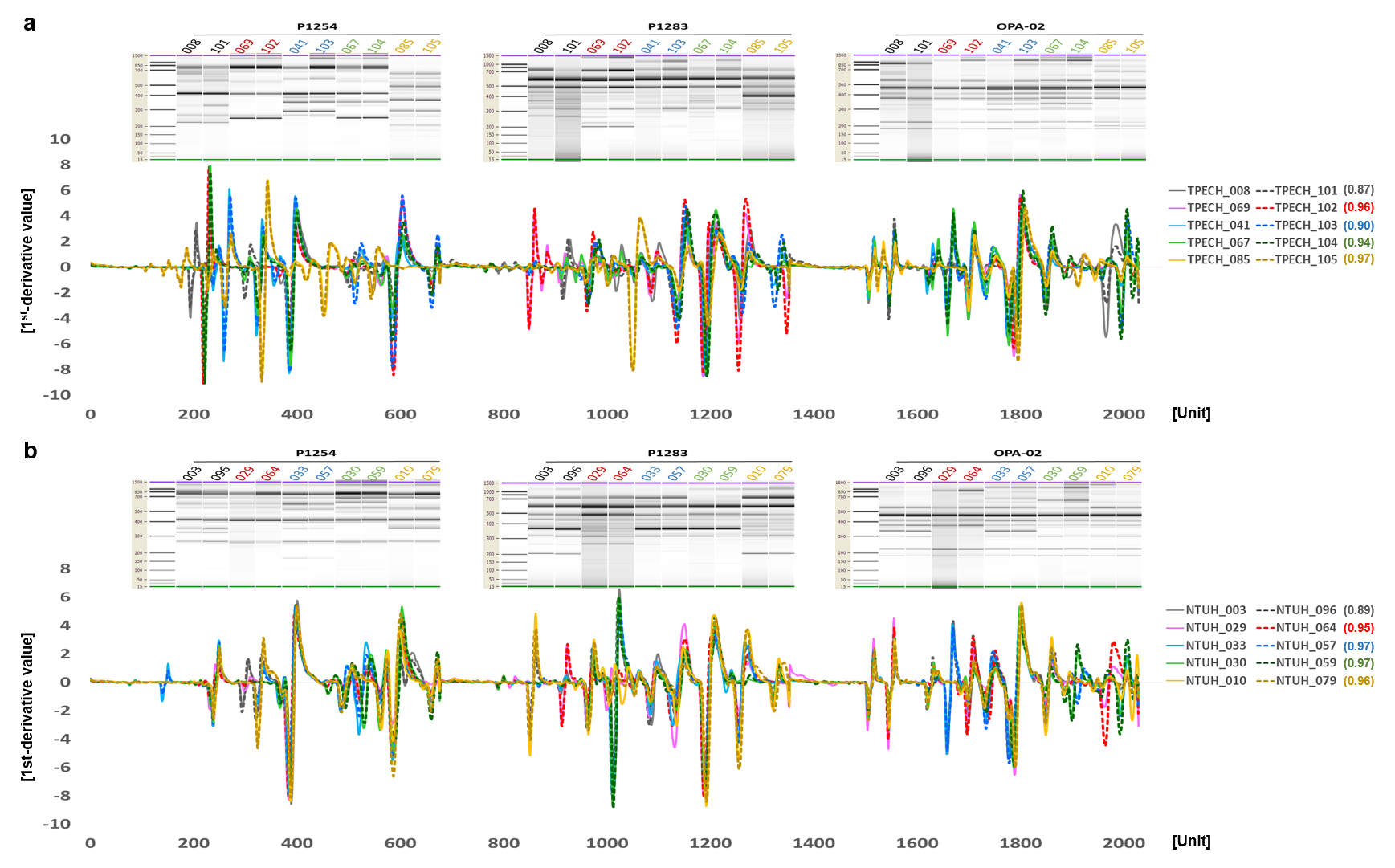


**Supplementary Figure S5.** **Confirmation of an extra amplicon in the CE-RAPD profile of TPECH_008/101.** (**a**) TPECH_008/101 and the NTUH_029 matched cluster samples were amplified using RAPD primer P1254. An extra amplicon from TPECH_008/101 is shown in the AGE (red arrow) and in the CE-RAPD profiles (pink box). (**b**) Specific PCR was used to detect the expression of integrase core domain protein (about 700 bp length) in ST11 group.


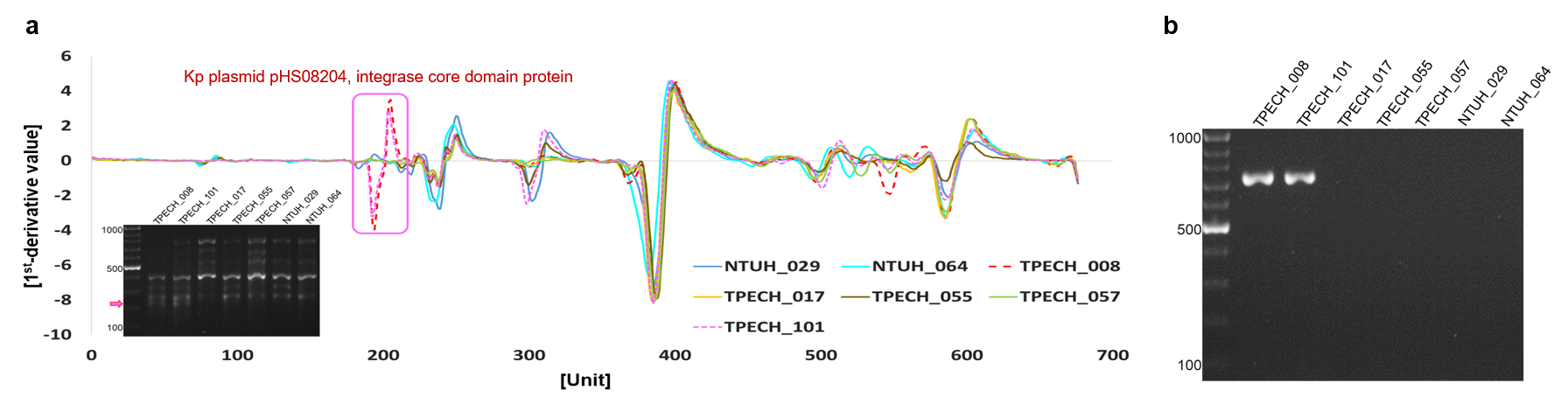


References

1. Daly, M. *et al.* Molecular characterization of Irish *Salmonella enterica* serotype Typhimurium: detection of class I integrons and assessment of genetic relationships by DNA amplification fingerprinting. *Appl Environ Microbiol* **66**, 614-619 (2000).
2. Patton, T. G., Katz, S., Sobieski, R. J. & Crupper, S. S. Genotyping of clinical *Serratia marcescens* isolates: a comparison of PCR-based methods. *FEMS Microbiol Lett* **194**, 19-25 (2001).
3. Abou-Dobara, M. I., Deyab, M. A., Elsawy, E. M. & Mohamed, H. H. Antibiotic susceptibility and genotype patterns of *Escherichia coli*, *Klebsiella pneumoniae* and *Pseudomonas aeruginosa* isolated from urinary tract infected patients. *Pol J Microbiol* **59**, 207-212 (2010).
4. Diancourt, L., Passet, V., Verhoef, J., Grimont, P. A. & Brisse, S. Multilocus sequence typing of *Klebsiella pneumoniae* nosocomial isolates. *J Clin Microbiol* **43**, 4178-4182 (2005).
